# Smoking Habits Among Patients Diagnosed with Oral Lichen Planus

**DOI:** 10.1186/1617-9625-2-9

**Published:** 2004-06-15

**Authors:** Meir Gorsky, Joel B Epstein, Haya Hasson-Kanfi, Eliezer Kaufman

**Affiliations:** 1 The Maurice and Gabriela Goldschleger School of Dental Medicine, Tel Aviv University, Tel Aviv Israel; 2 Department of Oral Medicine and Clinical Dentistry, College of Dentistry, and Interdisciplinary Program in Oral Cancer Biology, Prevention and Treatment, College of Medicine, Chicago Cancer Center, University of Illinois at Chicago, USA; 3 Department of Hospital Oral Medicine Hadassah Hebrew University, Faculty of Dental Medicine, Jerusalem, Israel; 4 Department of Oral Medicine and Diagnostic Sciences MC 838, 801 South Paulina Street, Chicago IL 60612 USA

## Abstract

**Introduction:**

Oral lichen planus (OLP) is one of the most common dermatologic diseases that manifests in the oral cavity. The purpose of this study was to evaluate the association between smoking habits and the clinical subtypes of OLP.

**Methods:**

Oral findings and smoking data from 187 charts of OLP patients from an oral medicine clinic was reviewed and compared to data from 76 matched control patients.

**Results and Discussion:**

Ninety-three patients were diagnosed with reticular OLP, 55 with atrophic and 39 with erosive forms of the disease. Symptomatic OLP occurred in 63.6% of patients. Fewer cases of reticular OLP were symptomatic than erosive OLP (p < 0.001). Significantly fewer OLP patients smoked than the control group (16% versus 25%) (p = 0.04). More patients with reticular OLP smoked than those with atrophic and erosive OLP (p = 0.002). It is hypothesized that the heat and irritation of smoking may aggravate symptomatic OLP lesions, and the risk of malignant transformation associated with tobacco use may play a role in patients stopping tobacco use. Because there were fewer smokers in patients with OLP, and because OLP carries an increased malignant risk, transformation of OLP may be due to a different etiology and of a different pathogenesis than squamous cell carcinoma not arising from lichen planus. Close follow-up of patients with OLP is indicated.

## Introduction

Lichen planus is a chronic inflammatory disease that involves skin and mucosa and is one of the most common dermatologic diseases that manifests in the oral cavity. The precise etiology of the disease is unknown, although it is well established that it involves immunologic processes. The prevalence of oral lichen planus (OLP) in the general population usually is 1%–2% [1,2], and OLP is found more commonly in women than in men [3,4].

Although the most common type of OLP seen in clinical settings is the erosive form (lesions that include erosions), which is almost always associated with pain [3,5], a higher prevalence of the reticular form (hyperkeratotic striae without erythemtous or erosive lesions) is reported in a previous study [4]. The reticular form of OLP is usually asymptomatic, although some patients may complain of an oral burning sensation or surface roughness. Patients with atrophic OLP (erythematous lesions without erosions) are more often symptomatic [4] and may have oral burning that is aggravated by spicy or acidic foods.

The association of tobacco smoking with OLP is not clearly understood. The onset of OLP has not been associated with smoking in some studies [3,5]. Neumann-Jensen and co-workers [6] stated that OLP was less common in smokers than in non-smoking patients. However, others have reported that OLP is more common in smokers [7]. Gorsky and associates [4] discussed the possibility of a correlation between different clinical manifestations of OLP and smoking, and it was felt that smoking was related to the severity of mucosal sensitivity associated with OLP. In a search of the English language literature, no additional study of smoking and the clinical presentation of OLP was identified. Gorsky and colleagues [4] speculated that the discomfort associated with the heat of the tobacco smoke accelerates mucosal pain in symptomatic OLP. It is possible that the discomfort associated with symptomatic OLP may play a role in the smoking patient's decision to stop smoking.

The purpose of the present study was to evaluate the association between smoking habits and the clinical subtypes of OLP and symptoms associated with OLP.

## Materials and methods

A total of 263 charts (187 patients with OLP and 76 non-OLP patients) from the Oral Medicine Clinic, School of Dental Medicine, Tel Aviv University, were reviewed. All patients completed standard institutional consent forms.

The study group included 187 patients (122 women, 65 men) seen between 1994 and 1999 with histologically confirmed OLP. Patients were evaluated by one clinician (MG). The clinical findings included reticular or papular white changes with or without erythema or erosion. The histologic criteria for diagnosis included hyperorthokeratosis and parakeratosis, degenerative changes of basal cells, the presence of civatte bodies and a band-like subepithelial infiltration of lymphocytes. The collected data included information regarding age, gender, signs and symptoms, sites of involvement and the clinical form of OLP (reticular, atrophic, erosive) [5]. Oral pain was reported on a four-point scale (0 = asymptomatic, 1 = mild, 2 = moderate, 3 = severe).

Seventy-six randomly selected charts of non-OLP and asymptomatic patients with different oral lesions (hyperkeratosis, fibromas, papillomas, melanotic macule), seen during the same time period, served as the control group. The two groups matched for age and sex.

Chi square and analysis of variance (ANOVA) were used to examine statistical significance.

## Results

The study group consisted of 122 (65.2%) females and 65 (34.8%) males with a mean age of 54.3 years (range 22 to 83 years) (Table 1). The control group included 45 (59.2%) females and 31 (40.8%) males with a mean age of 56.1 years (range 20 to 81 years). No significant difference was noted between the two groups in gender and age.

**Table 1:** Patient characteristics

	**Males **(%)	**Females **(%)	**Total**
OLP patients	65 (34.8)	122 (62.5	187

Control patients	31 (40.8)	45 (59.2)	76

**Figure 1:**
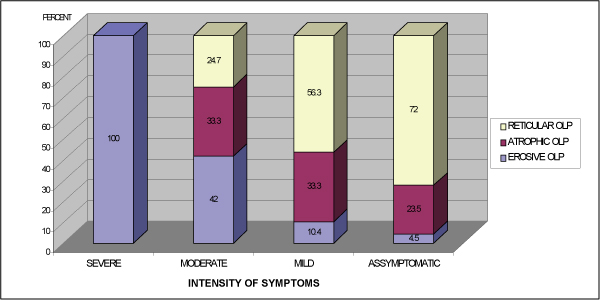
**The intensity of symptoms in subtypes of oral lichen planus**.

Ninety-three patients were diagnosed with reticular OLP, 55 with atrophic and 39 with erosive forms of the condition. The most prevalent site of involvement was the buccal mucosa (86%), followed by the gingiva (42.2%), tongue (34.7%), floor of the mouth (5.8%), lips (4.8%) and palate (3.7%). Approximately two-thirds of the OLP patients [119(63.6%)] were symptomatic and 68 (36.4%) patients had no symptoms at the initial clinic visit. Forty-nine (52.7%) patients with reticular lesions were "asymptomatic" and 27 (29%) patients reported mild symptoms. A significant correlation was found between reticular OLP and none or mild symptoms (p < 0.001) (Figure 1), and moderate or severe symptoms were associated with erosive OLP (p < 0.001). Thirty-one patients (79.5%) with erosive OLP reported oral symptoms. Severe symptoms were reported only by two patients, both with the erosive type of OLP (Figure 1). No statistical association was found for the atrophic form of OLP and the presence and intensity of symptoms.

**Figure 2:**
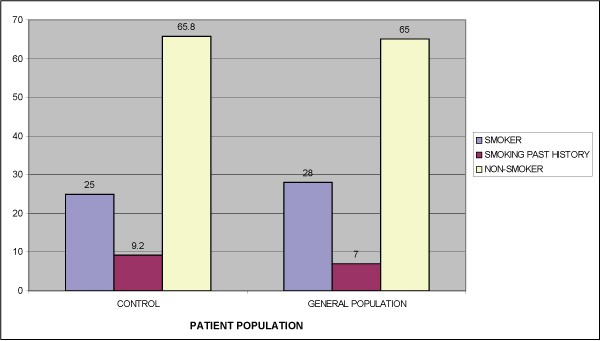
**Smoking habits in the control group and the general population (percentage)**.

No significant differences were noted between the smoking history of the control group and that of the general Israeli population [8] (Figure 2). No significant difference in smoking (combined current smokers and past smokers) was noted between the OLP patients (38.5%) and the control group (34.2%, p > 0.05) (Table 2). A significantly lower percentage of OLP patients (16%) smoked compared to control patients (25%) (p = 0.04). Based on the patients' reports, about one quarter (22.5%) of OLP patients discontinued smoking compared to less than 10% (9.2%) in the control group, shortly after changes in the oral mucosa were identified. The difference in smoking cessation between the two groups is statistically significant (p = 0.01). No significant difference in smoking (combined current smokers and past smokers) was noted between the two groups (38.5% in OLP patients and 34.2% in the control group; p > 0.05). Significantly more women than men reported never smoking (71.3% and 43% respectively; p = 0.02), although significantly more men stopped smoking (p = 0.02). There was no difference between males and females in the prevalence of those reporting smoking at their initial clinical visit for assessment of OLP (p > 0.05).

**Table 2:** Smoking habits of the study and the control groups

**Patients**	**No**.	**Smoking (%)**	**Quit Smoking (%)**	**Never Smoked (%)**
OLP patients	187	30/187 (16.0)	42/187 (22.5)	115/187 (61.5)

Control patients	76	19/76 (25.0)	7/76 (9.2)	50/76 (65.8)

No significant difference was seen in the smoking habits of symptomatic and asymptomatic OLP patients (Table 3). Also, no significant differences were noted in daily cigarette consumption and the duration of smoking in years between symptomatic and asymptomatic OLP patients. No significant differences were noted in comparing the other parameters of the symptomatic and asymptomatic OLP patients.

**Table 3:** Smoking habits in symptomatic and asymptomatic OLP patients

Patients	No.	Smoking (%)	Quit Smoking (%)	Never Smoked (%)
Symptomatic	119	18 (15.1)	24 (20.2)	77 (64.7)

Asymptomatic	68	12 (17.6)	18 (26.5)	38 (55.9)

Total	187	30 (16.0)	42 (22.5)	115 (61.5)

**Figure 3:**
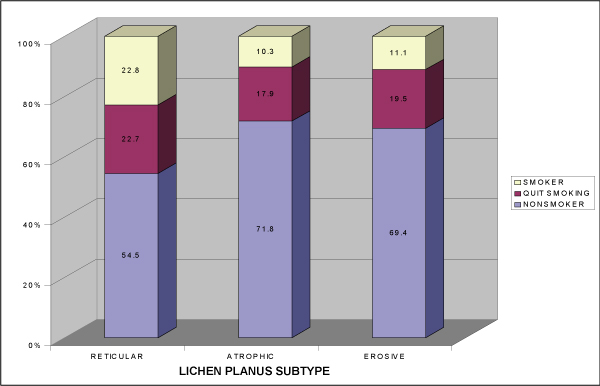
**Smoking habits in patients with symptomatic oral lichen planus**.

Almost 50% (49.7%) of the OLP patients were diagnosed with reticular OLP. Fifty-five patients (29.4%) had atrophic OLP and the remainder (20.9%) suffered from erosive lichen planus. Table 4 presents the smoking habits of patients with different forms of OLP. Differences in smoking habits between reticular OLP (23.7%) and atrophic (7.3%) or erosive OLP (10.3) were significant (p = 0.022). Significantly fewer patients with reticular OLP never smoked (50.5%) compared to each of the other subtypes of the disease (atrophic 72.7%, erosive 71.8%, p = 0.017). There were no significant differences in patients quitting smoking in the study groups. When the review of smoking habits of symptomatic OLP patients was examined (Figure 3), significantly more patients with reticular OLP had a history of smoking (45.5%) in contrast to the other forms of OLP (atrophic 28.2%, erosive 30.6%) (p = 0.02) and significantly more patients with reticular OLP were current smokers (p = 0.02).

**Table 4:** Smoking habits in the different clinical presentation of oral lichen planus

Smoking habits	Reticular (93)	Atrophic (55)	Erosive (39)
Smoking	22/93 (23.7%)	4/55 (7.3%)	4/39 (10.3%)

Quit smoking	24/93 (25.8%)	11/55 (20.0%)	7/39 (17.9%)

Never smoked	47/93 (50.5%)	40/55 (72.7%)	28/39 (71.8%)

## Discussion

The present study evaluated smoking in 187 patients with OLP. The mean age of the patients was beyond middle age. There were more females with OLP and there were more women with OLP who never smoked. The most prevalent site of occurrence was the buccal mucosa, followed by the gingiva and tongue. There was no difference between the genders in the prevalence of smoking at the time of the initial presentation. The higher prevalence of smoking cessation among OLP patients compared to control subjects may be related to aggravation of symptomatic OLP. This was discussed in a prior study, where it was suggested that the irritation of tobacco smoke in the oral cavity of an individual with OLP may further irritate the mucosal lesion [4]. In the current study, significantly fewer patients continued smoking after diagnosis of OLP (16%) than those in the control group (25%). The diagnosis of lesions with an increased risk of malignant transformation may also contribute to the increased quit rates reported in OLP patients.

No difference in smoking history was found between the control group and the general population (8), suggesting the representative nature of the control group in this parameter.

About one quarter (22.5%) of the OLP patients discontinued smoking compared to less than 10% (9.2%) in the control group. The smoking habits of symptomatic OLP patients were compared to those of the asymptomatic patients, and no difference in discontinuing smoking was noted. The general population is exposed to information concerning the risk factors and signs and symptoms of oral malignancies, which may lead patients to seek consultation for any suspicious changes in the oral mucosa. Changes in tobacco use may be due to recognition of oral mucosal lesions or irritation associated with smoking, and may reflect patient concerns and the benefits of public education.

In this study, 80% of patients with erosive OLP were symptomatic. Erosive OLP is the most symptomatic and the most prevalent subtype of OLP referred to oral medicine clinics [3,5]. Even though reticular OLP is the least symptomatic subtype of OLP, 50% of the patients in the present study were diagnosed with the reticular form of the disease. This finding is probably associated with the referral pattern of these patients, which may be a result of successful education of the general medical and the dental community.

Although there was no significant difference in smoking in patients with reticular OLP compared to the control group (23.7% and 25%), there were significantly more smokers in this group than in the two other subtypes of the disease (atrophic 7.3%, erosive 10.3%) (p = 0.022). This finding is not unexpected, since reticular OLP is significantly less symptomatic than atrophic and erosive OLP (p = 0.001), and smoking may not cause sensitivity of the oral mucosa in reticular OLP.

While malignant transformation of oral lichen planus remains controversial [9], recent studies of genetic change indicate that dysplastic OLP presents a molecular profile similar to that seen in other dysplastic oral lesions [10]. In addition, there is increasing evidence that up to 3.7% of cases progress to squamous cell carcinoma [3-5,11,12]. Furthermore, the erosive and atrophic forms of OLP may be at higher risk of malignant transformation [5,13]. Prior studies queried the potential impact of smoking on risk of progression of OLP to sqauamous cell carcinoma [14,15]. The present study found that the erosive and the atrophic forms of OLP are the least exposed to tobacco and suggests that malignant transformation may occur via different molecular pathways or due to different risk factors than in leukoplakia and erythroplakia or mixed lesions. The risk of malignant transformation in those patients diagnosed with OLP who are smokers may be associated with different risk factors and other pathways. Histopathologic evaluation for dysplasia and close follow-up is indicated in patients with OLP, particularly when dysplasia is identified.

The present study indicates that the smoking pattern in patients with OLP is different from that of the general population. Symptomatic OLP patients are more likely to discontinue smoking, likely due to increased tissue irritation. Tobacco use was more prevalent among patients with reticular OLP. Further studies on the influence of smoking behavior on the characteristics of symptomatic OLP and on the risk of malignant transformation of OLP are needed.

## Competing interests

The authors declare that they have no competing interests.
